# Supplementary figures and images for: Role of dietary sodium restriction in chronic heart failure: systematic review and meta-analysis
Source: Clin Res Cardiol. 2023 Jun 30;113(9):1331–42. doi: 10.1007/s00392-023-02256-7 (PMC11371846; doi:10.1007/s00392-023-02256-7)

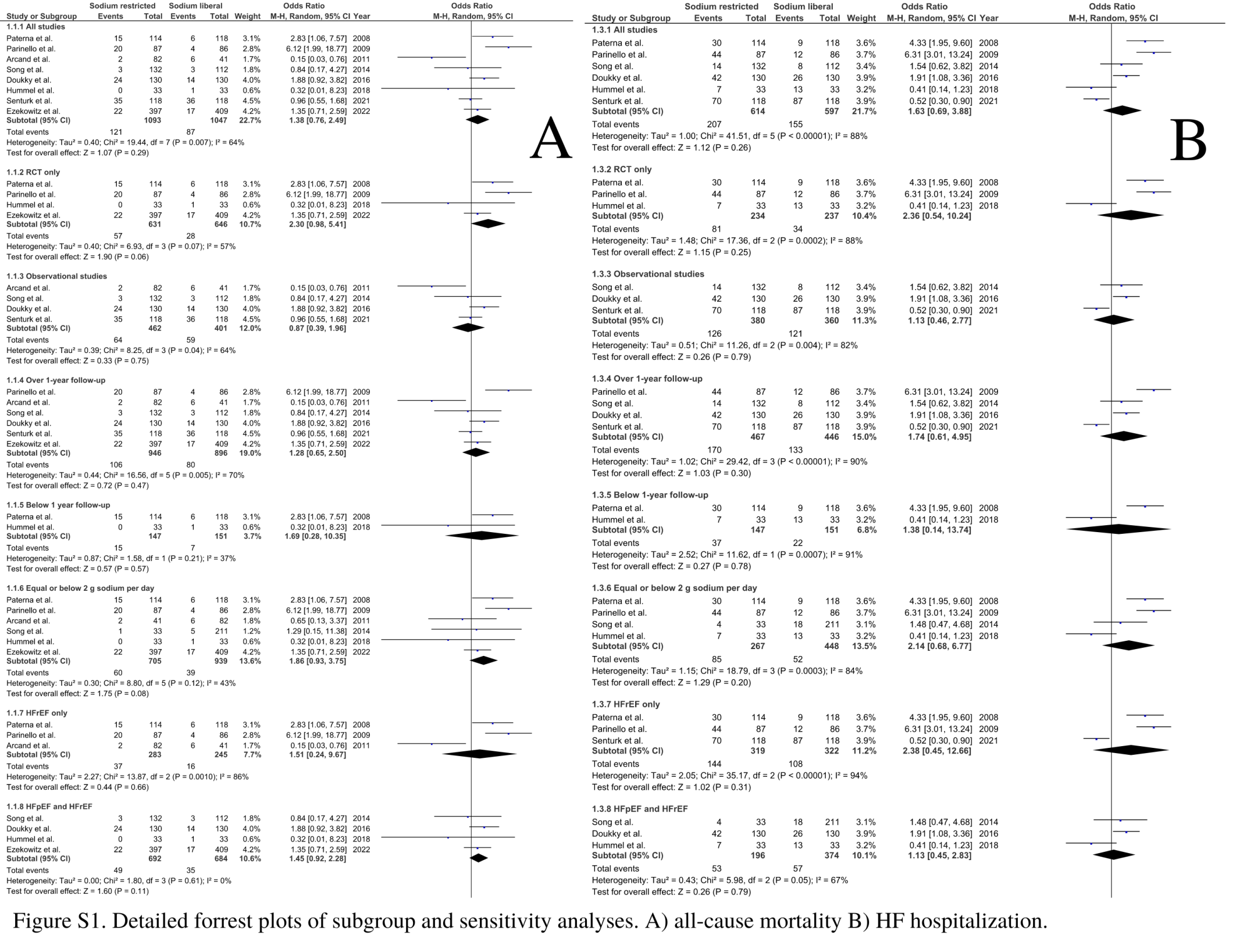

Supplement: Supplementary file 1 — Supplementary file1 (DOCX 1279 KB) [file 392_2023_2256_MOESM1_ESM.docx]
